# *Hepatozoon* (Eucoccidiorida: Hepatozoidae) in wild mammals of the Americas: a systematic review

**DOI:** 10.1186/s13071-024-06154-3

**Published:** 2024-03-06

**Authors:** Richard Thomas, Adriana Santodomingo, Liliana Saboya-Acosta, Julian F. Quintero-Galvis, Lucila Moreno, Juan E. Uribe, Sebastián Muñoz-Leal

**Affiliations:** 1 Departamento de Ciencia Animal, Facultad de Ciencias Veterinarias, Universidad de Concepción, Chillán, Chile; 2 Pontificia Universidad Javeriana, Facultad de Estudios Ambientales y Rurales, Doctorado en Estudios Ambientales y Rurales, Carrera 7 N 40-62, Bogotá, Colombia; 3 Instituto de Ciencias Ambientales y Evolutivas, Universidad Austral de Chile, Valdivia, Chile; 4 Millenium Nucleus of Patagonian Limit of Life (LiLi), Valdivia, Chile; 5 Departamento de Zoología, Facultad de Ciencias Naturales y Oceanográficas, Universidad de Concepción, Concepción, Chile; 6 Departamento de Biodiversidad y Biología Evolutiva, Museo Nacional de Ciencias Naturales (MNCN-CSIC), Madrid, Spain

**Keywords:** Arthropod-borne diseases, Hemogregarines, Conoidasida, Phylogeny, Haplotype diversity, Wildlife conservation

## Abstract

**Background:**

The study of parasites provides insight into intricate ecological relationships in ecosystem dynamics, food web structures, and evolution on multiple scales. *Hepatozoon* (Eucoccidiorida: Hepatozoidae) is a genus of protozoan hemoparasites with heteroxenous life cycles that switch infections between vertebrates and blood-feeding invertebrates. The most comprehensive review of the genus was published 26 years ago, and currently there are no harmonized data on the epizootiology, diagnostics, genotyping methods, evolutionary relationships, and genetic diversity of *Hepatozoon* in the Americas.

**Methods:**

Here, we provide a comprehensive review based on the PRISMA method regarding *Hepatozoon* in wild mammals within the American continent, in order to generate a framework for future research.

**Results:**

11 out of the 35 countries of the Americas (31.4%) had data on *Hepatozoon*, with Carnivora and Rodentia orders having the most characterizations. Bats, ungulates, and shrews were the least affected groups. While *Hepatozoon americanum*, *H. americanum*-like, *H. canis*, *H. didelphydis*, *H. felis*, *H. milleri*, *H. griseisciuri*, and *H. procyonis* correspond to the identified species, a plethora of genospecies is pending for a formal description combining morphology and genetics. Most of the vectors of *Hepatozoon* in the Americas are unknown, but some flea, mite, and tick species have been confirmed. The detection of *Hepatozoon* has relied mostly on conventional polymerase chain reaction (PCR), and the implementation of specific real time PCR for the genus needs to be employed to improve its diagnosis in wild animals in the future. From a genetic perspective, the V4 region of the 18S rRNA gene has been widely sequenced for the identification of *Hepatozoon* in wild animals. However, mitochondrial and apicoplast markers should also be targeted to truly determine different species in the genus. A phylogenetic analysis of herein retrieved 18S ribosomal DNA (rDNA) sequences showed two main clades of *Hepatozoon*: Clade I associated with small mammals, birds, and herpetozoa, and Clade II associated with Carnivora. The topology of the tree is also reflected in the haplotype network.

**Conclusions:**

Finally, our review emphasizes *Hepatozoon* as a potential disease agent in threatened wild mammals and the role of wild canids as spreaders of *Hepatozoon* infections in the Americas.

**Graphical Abstract:**

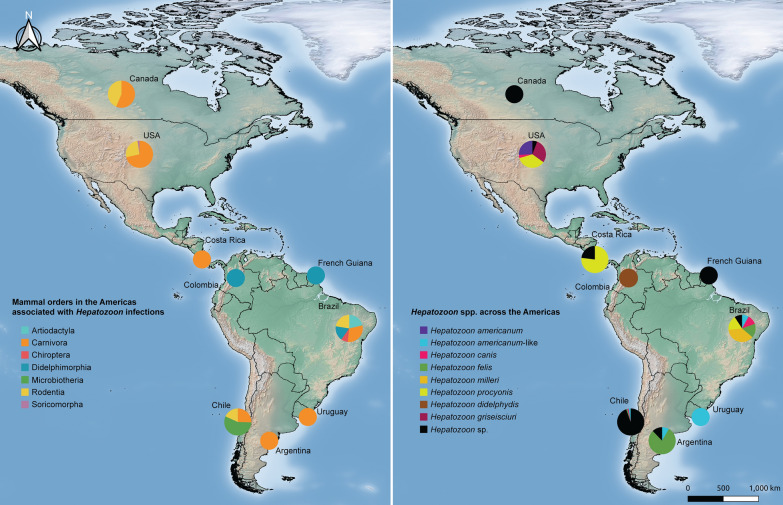

**Supplementary Information:**

The online version contains supplementary material available at 10.1186/s13071-024-06154-3.

## Background

The ongoing sixth mass extinction has driven efforts in documenting and understanding animal biodiversity for conservation and management [1]. Wild vertebrates and their parasites maintain infectious agents such as viruses, bacteria, and protozoa that may eventually spill over to humans or domestic animals, leading to outbreaks or epidemics [2–4]. As most species on earth are parasites, their role as indicators of the ecosystem’s health has gained attention in recent years [5, 6]. To complete biological cycles, parasites rely on the trophic interactions of their hosts [7]. Indeed, while some parasites co-evolve with their hosts, causing minimal damage, others are etiological agents [8–10]. This duality in the biology of parasites is important to understanding intricate ecological relationships, ecosystem dynamics, food web structure, and evolution across multiple scales [9, 10]. Additionally, infectious diseases caused by parasites pose significant conservation challenges due to their impact on wild animals [11, 12]. Therefore, identifying the parasites that act as etiological agents is crucial in defining the threats to wildlife conservation [13, 14].

*Hepatozoon* (Eucoccidiorida: Hepatozoidae) is a genus of protozoa that invades red and white blood cells of vertebrates [15, 16]. *Hepatozoon* spp. are commonly found in wildlife perpetuating in enzootic cycles [15, 17]. These hemoparasites have heteroxenous life cycles involving a vast array of intermediate hosts (vertebrates) and definitive hosts (blood-feeding invertebrates) [15, 16], a fact that has shaped the remarkable biological diversity of the genus.

After its original description by Miller in 1908, the most comprehensive review on the genus *Hepatozoon* was published 90 years later [15]. Further reviews added data mainly on canine hepatozoonosis [16, 18, 19], and there are currently no harmonized data on the epizootiology of the genus *Hepatozoon* in the Americas. Here we provide a comprehensive review, based on the Preferred Reporting Items for Systematic Reviews and Meta-Analyses (PRISMA) method [20], of *Hepatozoon* in wild mammals of the American continent, with the following objectives: (i) to catalogue the publications since the original description of the taxon, (ii) to compare the publications by country, (iii) to compile the nature of biological samples and the techniques employed to detect and genetically characterize the parasite, (iv) to assess the conservation status of wild mammals with confirmed infection, and (v) to analyze available genetic data. Collectively, the objectives of this review were to generate a framework of reference for future investigation on *Hepatozoon* in the Americas.

## Methods

### Literature search and database

We recovered literature on *Hepatozoon* published between 1916 and December 31, 2022, through an exhaustive review using reputable sources including PubMed (https://pubmed.ncbi.nlm.nih.gov/), SciELO [Scientific Electronic Library Online] (https://scielo.org/es/), Scopus (https://www.scopus.com/home.uri), and Web of Science (https://webofknowledge.com/UA). Only peer-reviewed publications were included. Data from books, gray literature such as scientific conferences, and theses were excluded. Data on *Hepatozoon* retrieved from domestic animals or invasive rodents were not quantified, but molecular sequences were included in genetic analyses.

To mitigate bias and maximize the number of available studies, we used targeted search strings and title-abstract-keywords unit searches, incorporating the terms “*Hepatozoon*,” “hepatozoonosis,” “wild mammals,” “wildlife,” “wild animals,” and “mammals,” and names of the countries in the American continent, along with names of orders and groups of mammals native to the Americas, as detailed in the *Handbook of the Mammals of the World* [21]. To hone our search, we applied Boolean operators “AND” and “OR,” and followed the advanced search protocols suggested in the selected databases.

Compiled papers were subjected to the PRISMA protocol [20], with the following inclusion criteria: (i) reports of *Hepatozoon* in mammals of the Americas and (ii) studies on ectoparasites extracted from mammals of the Americas that assessed the presence of *Hepatozoon*. The documents lacking these criteria were excluded. The metadata matrix included the publication year, country, vertebrate host order, family, species, type of sample, diagnostic technique, conservation and population statuses of the hosts according to the International Union for Conservation of Nature (IUCN) Red List (https://www.iucnredlist.org/), primers used in molecular diagnostics, and study references. Scientific names and taxonomy of the hosts species referenced in this review followed the Integrative Taxonomic Information System (ITIS) (https://www.itis.gov/).

### Analysis of sequences

#### Sequence selection and alignment

To define the final dataset included in this study, sequences of *Hepatozoon* and associated metadata were sourced from the GenBank database (https://ncbi.nlm.nih.gov/nucleotide) up to December 31, 2022, using “*Hepatozoon*” as the search criterion. Subsequently, the sequence filtering was carried out in four steps. First, only 18S ribosomal DNA (rDNA) sequences were included. Second, sequences annotated with a nomenclature other than *Hepatozoon* were excluded. Third, each sequence was individually aligned against an annotated 18S rDNA reference sequence of *Hepatozoon* (MH615006) to identify those sequences that showed more than 50% coverage for a given region of the 18S rRNA gene. Fourth, each sequence that satisfied the aforementioned criteria was recorded in a table, specifying the GenBank accession number, submission date, sequence length, and the flanked region of the 18S rRNA gene. Finally, all sequence datasets were aligned with MAFFT using the G-INS-i algorithm [22].

#### Phylogenetic analyses

Phylogeny was inferred using maximum likelihood (ML [23]) and Bayesian inference (BI [24, 25]) methods in IQ-TREE version (v.) 1.6.12 and [26] MrBayes v. 3.2.6 [27], respectively. The best ML and BI evolutionary models and phylogenetic reconstructions were calculated using the “-m TESTNEWONLY -mrate G” and “lset nst=mixed rates=gamma” commands, implemented in IQ-TREE [28] and MrBayes [27, 29], respectively. All best-fit models were selected under the Bayesian information criterion (BIC) [30].

Rapid hill-climbing and stochastic disturbance methods were employed for ML phylogenies, with 1,000 ultra-fast bootstrapping pseudo-replicates to evaluate the inferred tree robustness. Ultra-fast bootstrap values < 70%, 70–94%, and ≥ 95% were considered non-significant, moderate, and high statistical support, respectively [31]. Regarding the BI phylogenies, two independent tests of 20 × 10^6^ generations and four Markov chain Monte Carlo (MCMC) chains were implemented, sampling trees every 1,000 generations and removing the first 25% as burn-in. Tracer v. 1.7.1 [32] was employed to confirm the correlation and effective sample size (ESS) of the MCMCs. Bayesian posterior probabilities (BPP) > 0.70 at nodes were considered high statistical support [33]. Trees were visualized and edited with FigTree v. 1.4.1 (http://tree.bio.ed.ac.uk/software/figtree/) and Inkscape v 1.1 (https://inkscape.org/es/). A consensus phylogram for both ML and BI was generated following the approach outlined by Santodomingo et al. [34].

#### Haplotype analyses

A haplotype analysis of *Hepatozoon* genotypes was intended to evaluate genetic variants circulating among wild mammals in the Americas. According to the National Human Genome Research Institute (https://www.genome.gov/), a genotype is defined as a DNA sequence at a specific locus, whereas a haplotype is a set of genetic variants, or polymorphisms, that tend to be inherited together. To calculate the number of polymorphic sites (*S*), haplotypes (*H*), haplotype diversity (Hd), nucleotide diversity (*π*), and haplotype frequencies, the gametic phase was inferred using the PHASE module in DNAsp v. 6.12.03 software [35]. Nucleotide polymorphism analyses excluded gaps and considered invariable sites. A median-joining (MJ) haplotype network [36] was constructed using PopART v. 1.7 (http://popart.otago.ac.nz) to display haplotype frequencies.

### General findings

**Fig. 1:**
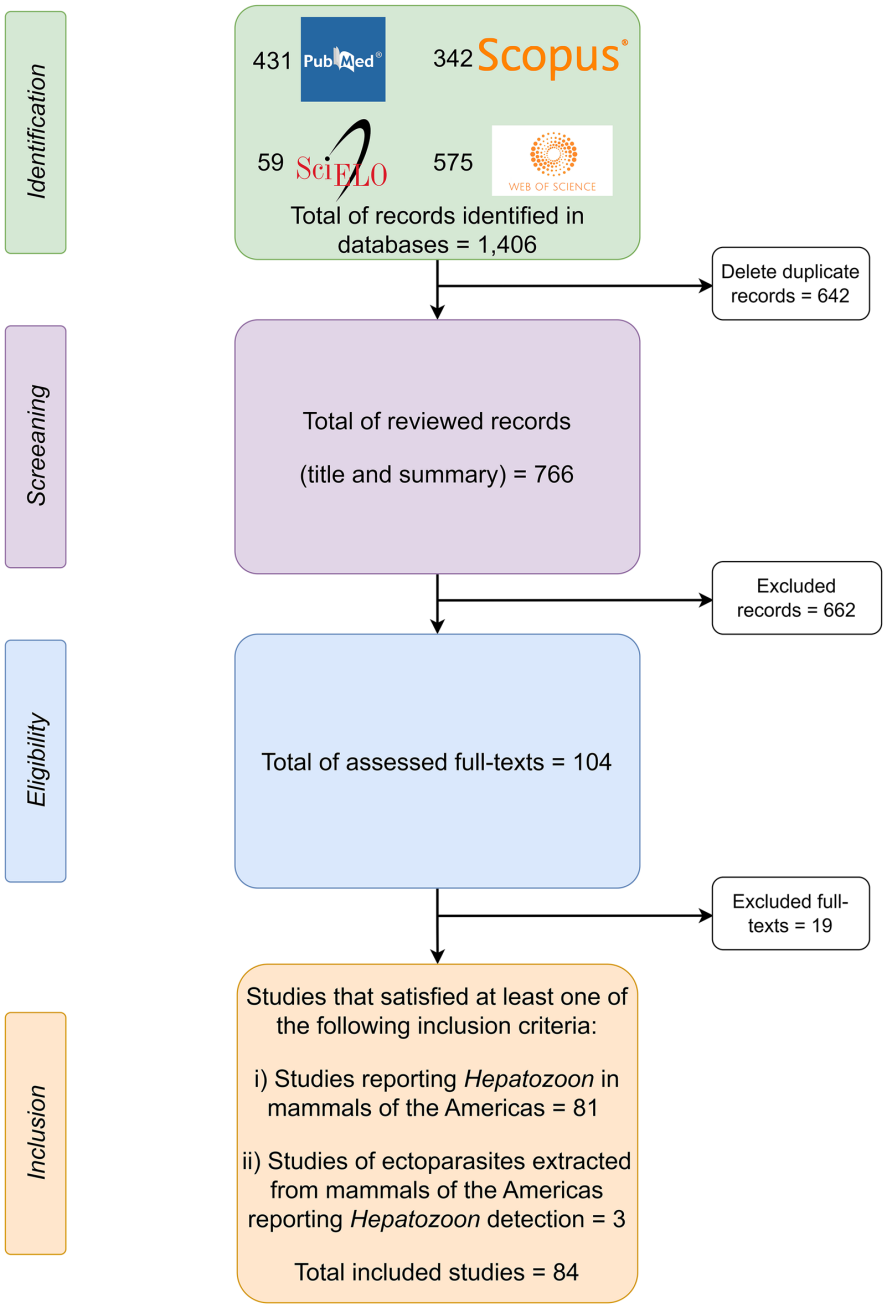
Flowchart of the current systematic review on Hepatozoon infections in wild mammals and their ectoparasites across the Americas (constructed with draw.io v. 22.0.3; https://www.drawio.com/)

A total of 1,406 studies were identified as potentially useful for the review. After thoroughly reviewing the papers meeting the selection criteria, 84 studies (5.97%) were selected, as they contained information on *Hepatozoon* in wild mammals in the Americas and their ectoparasites (Fig. 1). Eleven out of the 35 countries of the Americas (31.4%), including Argentina, Brazil, Canada, Chile, Colombia, Costa Rica, French Guiana, Panama, Uruguay, the USA, and Venezuela, had data on *Hepatozoon* (Additional file 1: Table S1). No data were found for the other 24 American countries.

The majority of studies on *Hepatozoon* in wild mammals were concentrated in Brazil and the USA, with 36 (42.9%) and 30 (35.7%) papers, respectively (Additional file 1: Table S1). In contrast, other countries in the Americas yielded less than five studies (~ 6%), underscoring a significant gap in the literature. In fact, research in the Americas has largely focused on domestic animals [16, 19, 37, 38]. This disparity not only underscores the need for a broader geographical scope in future studies, but also calls for assessing the occurrence of *Hepatozoon* in wild mammals in regions that currently lack data. In this regard, Chile displays a recent uptick in research on *Hepatozoon* infections in wildlife (Additional file 1: Table S1).

**Fig. 2:**
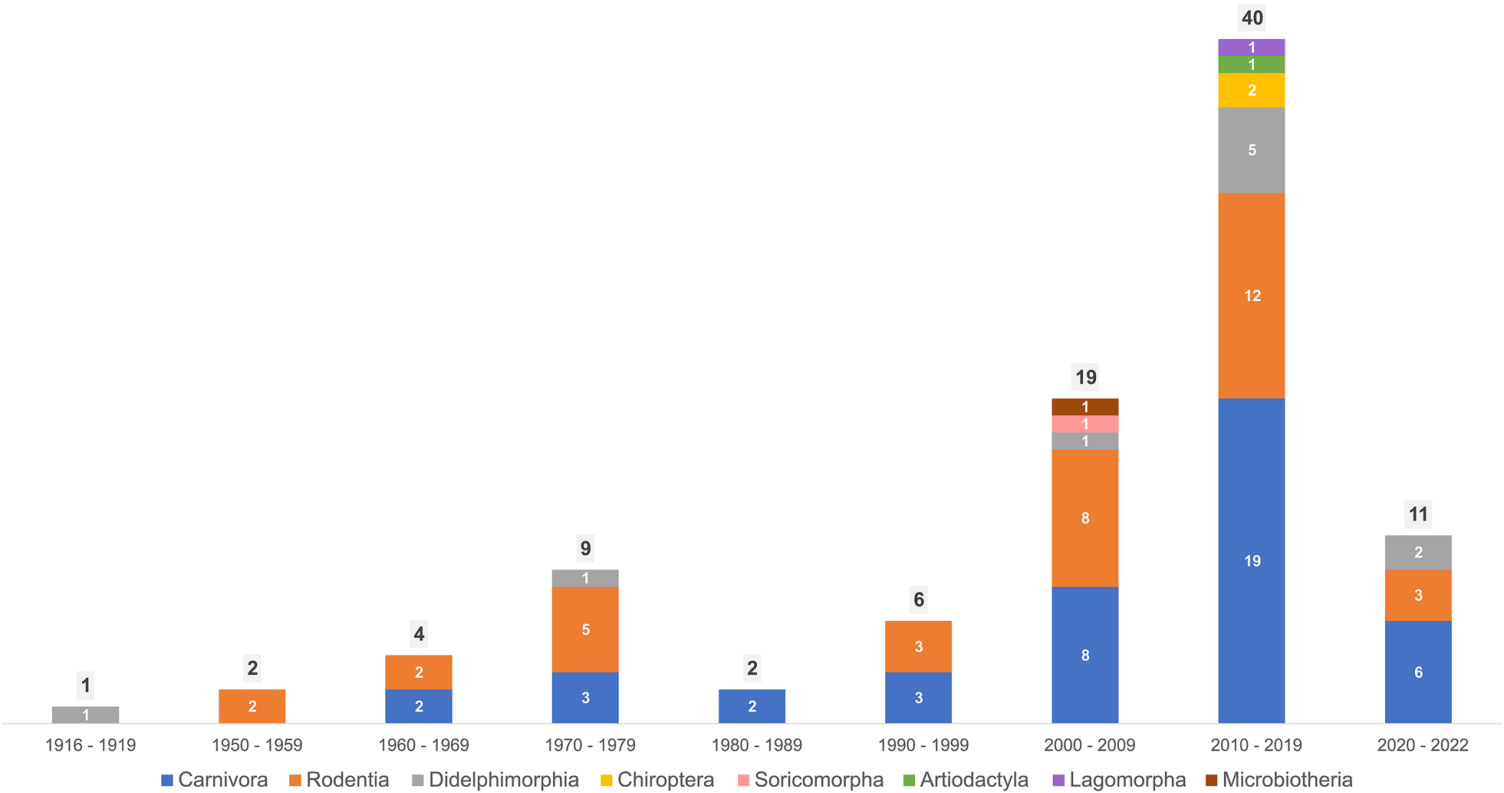
Temporal patterns of published articles regarding *Hepatozoon* organized by mammal orders in the American Continent

Regarding the number of studies on *Hepatozoon* among mammalian orders, Carnivora and Rodentia had the most, with 43 (51.2%) and 35 (41.7%) reports, respectively. Didelphimorphia followed, with 10 studies (11.9%), Chiroptera with two studies (2.4%), and Artiodactyla with two studies (2.4%). Lagomorpha, Microbiotheria, Perissodactyla, and Soricomorpha each had one study (1.2%) (Fig. 2). The disproportional distribution of studies by country—predominantly in Brazil and the USA—indicates a geographical bias. This fact not only skews the distribution of *Hepatozoon* spp., but also highlights the lack of a comprehensive understanding of infection by this protozoan in other mammalian orders. Furthermore, it denotes the limited research efforts on these hemoparasites in other American countries.

Overall, the number of studies on *Hepatozoon* in mammals of the Americas has displayed a notable upswing in recent decades (Fig. 2). Undoubtedly, the availability of molecular diagnostic tools contributed to this increase [39]. Indeed, molecular tools enhance sensitivity in detecting *Hepatozoon* from different types of biological samples, thus facilitating the identification of new species and previously unrecognized infections in wild mammals. The potential impact of these infections on wildlife health, coupled with the growing interest in arthropod-borne diseases, particularly those transmitted by ticks, has also contributed to the increase in research regarding *Hepatozoon* in wild mammals.

### Epizootiology of *Hepatozoon* in mammals of the Americas

*Hepatozoon* spp. thrive in enzootic cycles [15, 17], which are ecological systems involving vertebrate hosts, vectors, environmental components, and the critical community size of hosts required to maintain the infectious agent indefinitely [2, 40]. *Hepatozoon* spp. are sustained in both vertebrate and invertebrate hosts [15], with transmission pathways that shift depending on the specific parasite-host interactions.

In vertebrates, the transmission routes of *Hepatozoon* primarily include the ingestion of infected ectoparasites containing mature oocysts, often during grooming [15, 16, 41, 42], and, to a lesser extent, the consumption of infected prey tissue carrying meronts or cystozoites (predation) or ectoparasites attached to their prey [43–46]. In mammals, transmission of macromeronts also occurs through the placenta (transplacental) [47–49]. However, this route was only confirmed in *Hepatozoon canis*, but it probably also occurs in *Hepatozoon americanum* [50]. Conversely, in ectoparasites, transstadial perpetuation has been documented only for *H. canis* [51] and *H*. *americanum* in ticks [52]. Although transovarial transmission has not been demonstrated for the *Hepatozoon* genus [51, 53, 54], the documented transmission strategies highlight the evolutionary success of *Hepatozoon* to complete life cycles.

*Hepatozoon* spp. infect a wide range of vertebrate hosts, including herpetozoa [15, 17, 55], birds [56, 57], and mammals [58, 59]. Additionally, blood-sucking arthropods such as flies, triatomines [15, 17, 60], mosquitoes [55, 61], ticks [52, 62, 63], fleas, lice, and mites [15, 64, 65] serve as vectors [15]. A total of 6,631 mammals have been analyzed in 81 studies across the American continent, with 1,789 animals testing positive for *Hepatozoon*, yielding a cumulative infection frequency (IF) of 26.93%. Notably, *Hepatozoon* IFs varied across mammalian orders (see Additional file 1: Table S1). Microbiotheria and Carnivora exhibited the highest IFs, with 86.67% (65/75) and 42.12% (730/1,733), respectively. Didelphimorphia had an IF of 25.95% (144/555), while Rodentia had an IF of 22.87% (798/3,489). Chiroptera displayed an IF of 11.21% (12/107), whereas Artiodactyla had an IF of 5.42% (33/609). In contrast, Soricomorpha had a remarkably low IF of 1.69% (1/59). Unfortunately, the sample sizes for the orders Lagomorpha and Perissodactyla were too small to provide a significant IF (Additional file 1: Table S1).

To date, 107 species of mammals from nine orders have been screened for *Hepatozoon*. Of these, two species of carnivores were found infected with *H*. *americanum* (1.89%) and five species with *H*. *americanum*-like (4.72%) (Additional file 1: Table S1). The former species is known for its virulence in canids [66]. The latter was considered an emerging South American variant of *H. americanum* [67]; however, *H. americanum*-like exploits a niche with different vectors and hosts, so it should be considered a different species. Moreover, pathogenic effects of *H. americanum*-like still need to be assessed in detail. In contrast, *H. canis*, which appears well adapted to its canine hosts [66, 67], has also been found infecting nine out of ten canid species in the continent (80%). Notably, some species of Artiodactyla, Chiroptera, Didelphimorphia, and Rodentia orders may also be susceptible to *H. canis* (Additional file 1: Table S1), a fact that underlines the generalist and opportunistic nature of this species.

*Hepatozoon felis*, the causative agent of feline hepatozoonosis [68], has been identified in six felids (5.66%), four canids (3.77%), two procyonids (1.96%), and one mustelid (0.94%) (Additional file 1: Table S1). *Hepatozoon felis* is the predominant species of *Hepatozoon* infecting wild felids worldwide [68]. Therefore, it is not unexpected to observe a higher IF in felids (49/158; 31.01%) compared to other animals (83/317; 26.18%) (Additional file 1: Table S1). However, the IF of *H*. *felis* in non-felid species may suggest that the hemoparasite could vary in host specificity, possibly due to transmission by ubiquitous vectors (such as flea, mite, or tick) or carnivorism among mammal groups [68]. These findings also raise questions about the potential shift that transmission dynamics of feline hepatozoonosis could undergo given the current population decline of South American felids (see Additional file 1: Table S1) [69].

A total of 60 mammal species (56.60%) were found to be infected with *Hepatozoon* spp. such as *Hepatozoon didelphydis*, *H*. *griseisciuri*, *H*. *milleri*, and *H*. *procyonis*, each one exhibiting a different IF and infecting specific mammal groups (as shown in Table S1). Furthermore, lineages of *Hepatozoon* have been documented, covering species associated with carnivores, herpetozoa and small mammals, opossums, reptiles, and rodents (Additional file 1: Table S1). According to Dupré [70], a “lineage” is defined as an independent evolutionary line that extends back in time from a current species to its ancestors. In this sense, *Hepatozoon* lineages reflect unique evolutionary histories and specific adaptations of *Hepatozoon* spp. to different hosts, suggesting putatively novel species associated with a diverse array of wild mammals in the Americas.

According to the data gathered (Additional file 1: Table S1), the IF of *Hepatozoon* spp. varies among mammals. Mustelids (Carnivora: Mustelidae) had the highest IF of 57.9% (11/19), followed by raccoons with 46.48% (317/682) and canids with 41.54% (339/816). Opossums showed an IF of 33.17% (209/630), while felids had lower IF of 29.17% (63/216). Regarding rodents, Sciuromorpha (Sciuridae) had the highest IF of 50.10% (247/493), while Hystricomorpha (Caviidae, Cuniculidae, and Echimyidae) and Myomorpha (Cricetidae) had IF of 31% (137/442) and 16.14% (412/2,552), respectively. Bats, ungulates, and shrews were the least affected groups, with IFs of 11.21% (12/107), 5.56% (34/611), and 1.7% (1/59), respectively.

Regarding specific *Hepatozoon* spp., *H. milleri* had the highest IF of 90.9% (10/11), followed by *H. griseisciuri* with 49.80% (244/490), *H. americanum* with 44.1% (41/93), and *H. procyonis* with 47.97% (296/617). In contrast, *H. didelphydis*, *H. canis*, and *H. americanum*-like had low IFs of 24.7% (23/93), 20.24% (133/657), and 11.43% (44/385), respectively. It is important to note that the small sample size may have biased the IF in *H*. *milleri*, and a similar reason could explain the high IF of *Hepatozoon* in mustelids.

### Blood-sucking arthropods associated with the epizootiology of *Hepatozoon*

Ticks, fleas, lice, and mites have been suggested as potential vectors of *Hepatozoon* spp. in mammals [15]. For instance, *Hepatozoon* DNA has typically been detected in mammal-associated ticks of the genera *Amblyomma* [71, 72], *Dermacentor* [73, 74], *Haemaphysalis* [62, 72, 73], *Ixodes* [75–79], and *Rhipicephalus* [42, 80]. Although the detection of *Hepatozoon* DNA in blood-sucking arthropods does not definitively prove any role in transmission [54], ticks harboring *Hepatozoon* DNA should not be ruled out as potential vectors [79].

Currently, several tick species, including *Amblyomma ovale* [81, 82], *Haemaphysalis longicornis*, *Haemaphysalis flava* [62], *Ixodes ricinus* [54], *Rhipicephalus microplus* [80], and *Rhipicephalus sanguineus* group [42, 53, 83], have been identified as vectors for *H. canis*. Additionally, *Amblyomma maculatum* is a recognized vector for *H. americanum* [52, 63, 84–86]. These ticks are frequently found on carnivores and ruminants, and their role in the spread of *Hepatozoon* spp. towards other mammal groups remains unclear.

Concerning mammal species in the Americas, blood-sucking ectoparasites that could be related to the epizootiology of the *Hepatozoon* species include ticks such as *Amblyomma dubitatum*, *A. maculatum*, *A. tigrinum*, and *A. sculptum* in carnivores [13, 71, 87–89]; *Ixodes neuquenensis* in Microbiotheria [75]; and *Amblyomma fuscum* and species of the *Ixodes sigelos* group in rodents [79, 90]. To better understand the implications of these ticks in the *Hepatozoon* epizootiology among the Americas’ ecosystems, experimental transmission studies are necessary to clarify these associations. Transstadial detection of oocysts in tick hemolymph would also contribute to elucidate vector roles.

While fleas, lice, and mites are also considered vectors or definitive hosts of *Hepatozoon* spp. associated with rodents [64, 65], only one flea species (*Megabothris abantis*) and two mite species (*Euhaemogamasus ambulans* and *Echinolaelaps echidninus*) have been reported as vectors for *Hepatozoon* spp. in rodents of the Americas [64]. Moreover, the fleas *Amalaraeus dissimilis* and *Peromyscopsylla ostsibirica* are possible vectors of *Hepatozoon* in rodents of the genus *Microtus* in Alaska [91]. The limited knowledge on *Hepatozoon* vectors stems from the challenges that the detection of *Hepatozoon* oocysts in blood-sucking arthropods pose [64, 92]. These may include finding the ectoparasites on their hosts, maintaining the vectors alive in laboratory conditions, and submitting their hemolymph to microscopical analyses for the detection of oocysts and sporocysts, and eventually genetic identification.

### *Hepatozoon* and concurrent infections

*Hepatozoon* infections can occur simultaneously with other infectious diseases and are common in regions where vector-borne diseases prevail. Blood-sucking arthropods are known to transmit a plethora of pathogenic bacteria and protozoa [93]. In the Americas, *Hepatozoon* infections in wild mammals have been observed alongside other infectious agents, including some with zoonotic potential. For example, concurrent infections of *Hepatozoon* with Piroplasmida spp. [94], *Rangelia vitalii*, *Leishmania* sp., [95, 96], *Anaplasma* sp., *Babesia* sp., and *Ehrlichia* spp. [97, 98] have been found in canids. Rodents have shown co-infections with *Babesia* spp., *Trypanosoma* sp. [99–102], *Anaplasma* sp., *Bartonella* spp., *Ehrlichia* spp., and *Theileria* sp. [98], while detections in felids point to co-infections with *Cytauxzoon felis*, *Cytauxzoon* sp., Piroplasmida sp., and *Theileria* sp. [94, 98, 100].

**Fig. 3:**
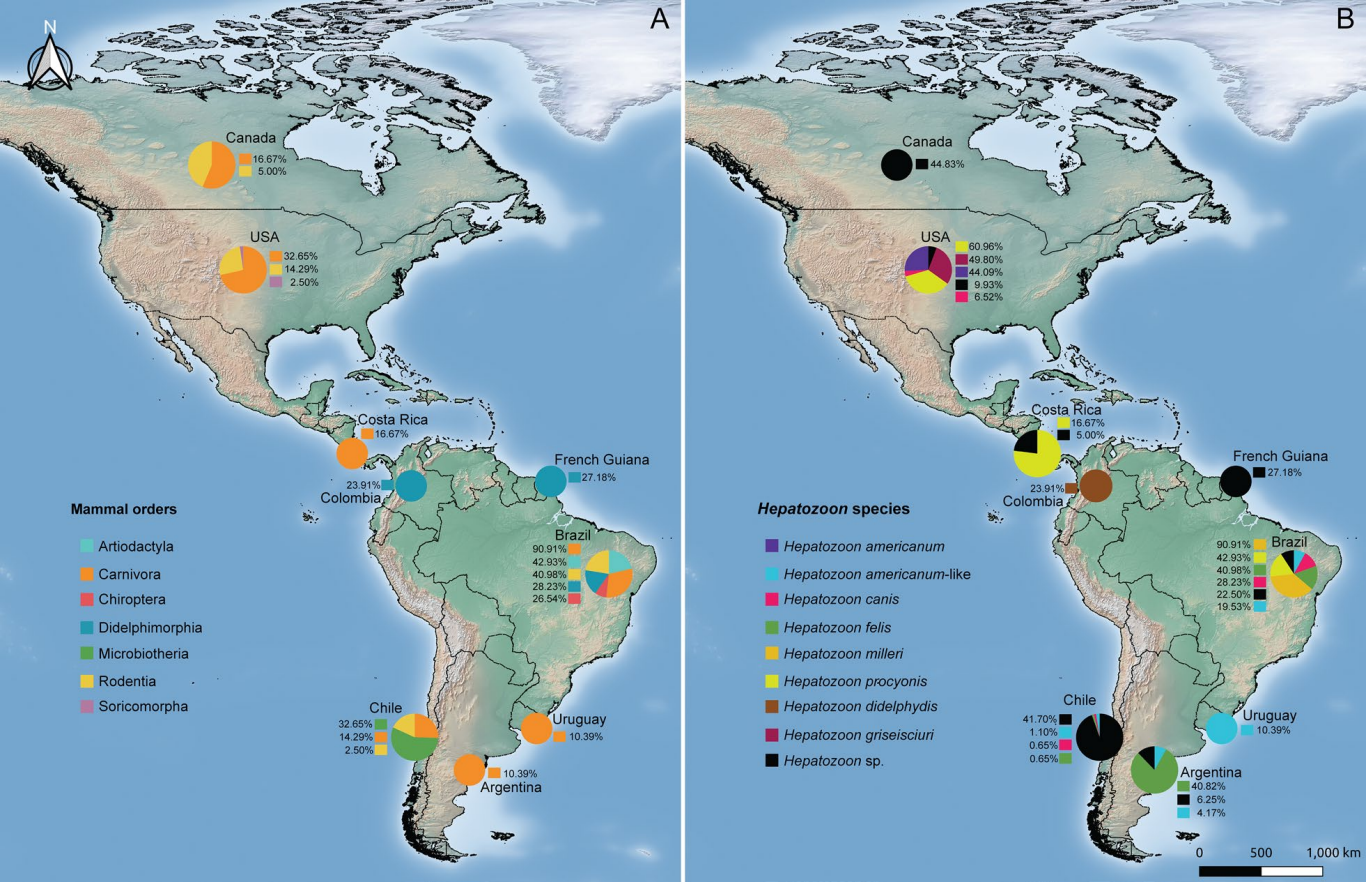
Map of the American Continent showing the infection rates (IRs) of *Hepatozoon* by mammalian order (A), and occurrence of *Hepatozoon* spp. in American country (B). Maps were constructed with Quantum Geographic Information System (QGIS) v. 3.18.1- Zürich (https://www.gnu.org/licenses)

Additionally, procyonids have shown co-infections with *Babesia microti* [103], *Trypanosoma cruzi* [104–106], *Anaplasma* sp., *Babesia* sp., *Ehrlichia* spp., and *Theileria* sp. [98], while opossums have concurrent infections with *Babesia* spp., *Ehrlichia* spp., Piroplasmida sp., and *Theileria* sp. [94, 98, 107, 108]. On the other hand, tapirids have been reportedly co-infected with *Theileria* sp. exclusively [94]. Furthermore, when *Hepatozoon* infections co-occur with other infections, they may lead to exacerbation of pre-existing conditions, resulting in severe morbidity, prolonged duration of clinical manifestations, and interference between diagnosis and treatment [92, 94, 108, 109]. Overall, the IF of *Hepatozoon* in mammals in the Americas varies between studies, as shown in Additional file 1: Table S1. The IFs of *Hepatozoon* in each mammalian order (Fig. 3A) and the IF of *Hepatozoon* spp. are illustrated by country (Fig. 3B).

### Detection and characterization of *Hepatozoon* in mammals of the Americas

#### Biological samples used to detect *Hepatozoon*

Blood was the main sample employed to detect and characterize *Hepatozoon*, with 56 studies (66.7%). The liver was the second most frequently used sample, accounting for 13 studies (15.5%), followed by the spleen with 12 studies (14.3%). Other samples included lung (10 studies; 11.9%), heart (eight studies; 9.5%), and muscle (seven studies; 8.3%). Less commonly employed samples were bone marrow, tail, skeletal muscle, kidney, and synovial fluid (ranging from 1.2% to 3.6%) (Additional file 1: Table S1). Moreover, as ticks feed on vertebrate blood, they were used as sentinels to assess the presence of *Hepatozoon* in wild mammals in three studies (3.6%) [78, 79, 90]. Therefore, the variety of tissues that *Hepatozoon* spp. may attain in the vertebrate host, including their ectoparasites, maximizes the chance of detection.

Detecting gamonts in blood is frequently used as a quick diagnosis of *Hepatozoon* infection in vertebrates [39]. However, the spleen is more sensitive, because it harbors meronts (groups of meront cells in multiple division) and subsequently higher loads of parasites; therefore, it is considered the best target for detection [42, 109]. Meronts have also been observed in bone marrow of red foxes (*Vulpes vulpes*) [48, 110]. Depending on the *Hepatozoon* species, alternative samples for detection and histopathology include biopsies of bone marrow or skeletal muscle for *H. canis* [66, 83, 111], skeletal muscle for *H. americanum* and *H. felis* [66, 68], and cardiac muscle for *H. felis* [68].

#### Diagnostic techniques to detect *Hepatozoon*

The most frequently used methods were conventional polymerase chain reaction (cPCR), employed in 47 studies (56%), blood smear in 28 studies (33.3%), and histology in 26 studies (30.9%). In contrast, nested PCR (nPCR) was less frequently employed, accounting for 4.8% (four studies), while bone marrow smear and leukocyte-platelet layer smear were used in two studies (2.4%) and one study (1.2%), respectively (Additional file 1: Table S1). The effectiveness of each technique varies depending on the intensity of infection. For instance, while observing blood smears is sensitive in animals with high parasitemia, histology is valuable in detecting subclinical infections, in which *Hepatozoon* encysts in different organs and fewer gamonts circulate in blood [39]. This is particularly relevant in *H. americanum* and *H. americanum*-like infection, two species that produce very low parasitemia during the clinical disease [16, 39, 84]. PCR stands out as the gold standard for diagnosing and characterizing *Hepatozoon* spp., particularly in cases of subclinical infection with low parasitemia [39, 112, 113], where nPCR can significantly improve the specificity [39, 89]. Although real-time PCR is highly sensitive, none of the reviewed studies implemented this technique. Applying real-time PCR to surveil *Hepatozoon* infections in wildlife could significantly expand diagnostic capabilities. This approach not only will enable the rapid processing of large sample volumes, reducing the time and costs of diagnosis, but also will improve the sensitivity of cPCR in detecting *Hepatozoon* infections [114, 115].

#### *Hepatozoon* genotyping

Molecular analyses of the phylum Apicomplexa have provided valuable insight into the genomic composition and genetic structure of the group, revealing the existence of three kinds of genomes: nuclear, mitochondrial, and the apicoplast [116–118]. The first genetic characterizations of the genus *Hepatozoon* relied on partial sequences of the apicoplast-encoded 16S rRNA gene [119], the nuclear ribosomal DNA internal transcribed spacer 1 (ITS-1) [120, 121], and the 18S rRNA gene [58, 59, 122, 123]. Since then, 18S rDNA sequences have been widely used for genotyping and molecular systematics of the genus [124–126].

Extrachromosomal genomes are often detected in significant quantities, with two or even 15 copies per cell [127, 128]. For the genus *Hepatozoon*, the first assembled mitochondrial genome (mitogenome) belonged to *Hepatozoon catesbianae*, a species that infects the frog *Lithobates catesbeianus* [129]. Moreover, the apicoplast genome of *H*. *canis* [128] and the mitogenome of *Hepatozoon* spp. associated with rodents were recently sequenced [125, 126]. It was not unexpected that the mitochondrial cytochrome *c* oxidase I (*cox1*) locus had a higher nucleotide divergence (≥ 0.08) if compared with the 18S rRNA gene (≥ 0.012), becoming the marker of choice to distinguish *Hepatozoon* species [125, 126, 128]. Nevertheless, the paucity of mitochondrial and apicoplast sequences in online databases represents a significant gap that needs to be addressed in order to unravel the intricate diversity and systematics of the genus. In this sense, the works of Léveillé et al. [125, 128, 129] and Hrazdilová et al. [126] are groundbreaking on a global scale.

Only one out of 84 reviewed articles used primers targeting the mitogenome of *Hepatozoon* in wild rodents [125]. In contrast, the remaining studies employed primers for the 18S rRNA gene to detect and characterize *Hepatozoon*. This approach has been the primary method applied in mammals of the Americas since 2006 (Additional file 1: Tables S1, S3). Overall, the 18S rRNA gene is conserved and frequently used in *Hepatozoon* phylogenies because it yields good resolution to the genus or even species level in the Apicomplexa order [123, 124, 126, 130]. Furthermore, the most abundant collection of *Hepatozoon* sequences in public database derives from this marker (Additional file 1: Tables S2, S3).

**Fig. 4:**
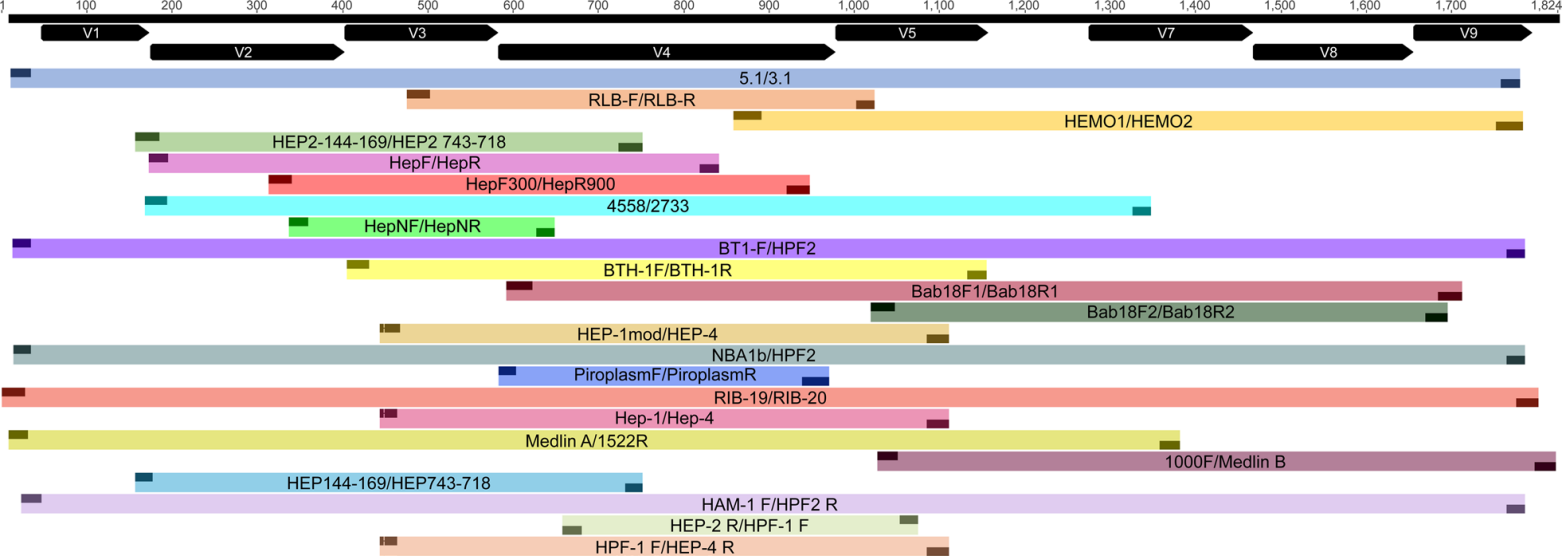
Primers and flanked regions (V1 to V9) used for the molecular diagnosis and genotyping of *Hepatozoon* in wild mammals in the Americas. The position of each primer was aligned with the 18S rDNA complete reference sequence of *Hepatozoon canis* detected in a domestic dog from Israel (MH615006, 1,816 bp; shown in black). For details on the primers, refer to Table S3

It is important to keep in mind that an accurate molecular detection of *Hepatozoon* spp. relies mostly in the selection of primers and an optimal thermal cycling condition [39]. Reported sequences in the reviewed studies were obtained through different PCR protocols, using 23 distinct primer sets (refer to Fig. 4 and Additional file 1: Table S2 for details). These protocols yielded sequences ranging from 300 to 1,816 base pairs (bp), with most primers targeting the V4 region of the 18S rRNA gene (Fig. 4).

A correct choice of primers for amplifying specific DNA fragments is crucial to achieving the objectives in a given study [39, 131]. A key aspect in primer selection is using those primers with the lowest potential to form secondary structures, such as hairpins or dimers (Additional file 1: Tables S2). For molecular diagnostics, it is preferable to opt for primers that amplify shorter fragments to maximize the sensitivity of the results [39]. Conversely, when conducting phylogenetic inferences, it is recommended to choose primers that yield longer fragments or even a complete gene sequence (Fig. 4, Additional file 1: Table S2) [39]. Nevertheless, some studies constructed phylogenies of the genus *Hepatozoon* incorporating short and long sequences, and the trees yielded congruent topologies consistent with the evolutionary history of the genus [124, 130, 132, 133]. It is important to note that these studies implemented short sequences above 387 bp flanking the IV region of the 18S rRNA gene.

### Evolutionary relationships and haplotype diversity of *Hepatozoon* spp. in mammals of the Americas

The small subunit of the ribosomal RNA gene (SSU rRNA or 18S rRNA) encompasses nine regions (V1–V9), with the regions V2, V4, and V9 the most hypervariable [131]. As of December 31, 2022, the GenBank database lists over 2,916 18S rDNA sequences (≥ 387 bp) associated with *Hepatozoon*, flanking one or more 18S rRNA gene variable regions (Additional file 1: Table S3). For the V4 region, 2,596 sequences (390–1816 bp) showed over 50% coverage, and 247 have been recovered from 48 wild mammal species across the Americas (Additional file 1: Table S3). Conversely, *Hepatozoon* sequences corresponding to other regions of the gene were less represented (Additional file 1: Table S3). Therefore, analyses of genotypes incorporated a final curated dataset of 115 sequences obtained from mammals in the Americas, along with 126 *Hepatozoon* sequences recovered from birds, small mammals, herpetozoa, and carnivores worldwide. In contrast, haplotype analyses were confined to the aforementioned 115 sequences. All sequences overlapped in the V4 region of the 18S rRNA gene, each with ≥ 500-bp length, similar to the approach applied by Vásquez-Aguilar et al. [38].

Before detailing the results of genotype and haplotype analyses, a brief recall on the definitions of these terms is given for clarity of interpretation. A genotype refers to the specific DNA sequence of a given locus, while a haplotype encapsulates a collection of genetic variants, typically co-inherited. These definitions are instrumental to analyze and understand the phylogenetic relationships and genetic variability within the *Hepatozoon* genus, enabling a deeper comprehension of the evolutionary dynamics shaping the species among diverse ecological niches.

### Phylogenetic inferences and haplotype diversity

**Fig. 5:**
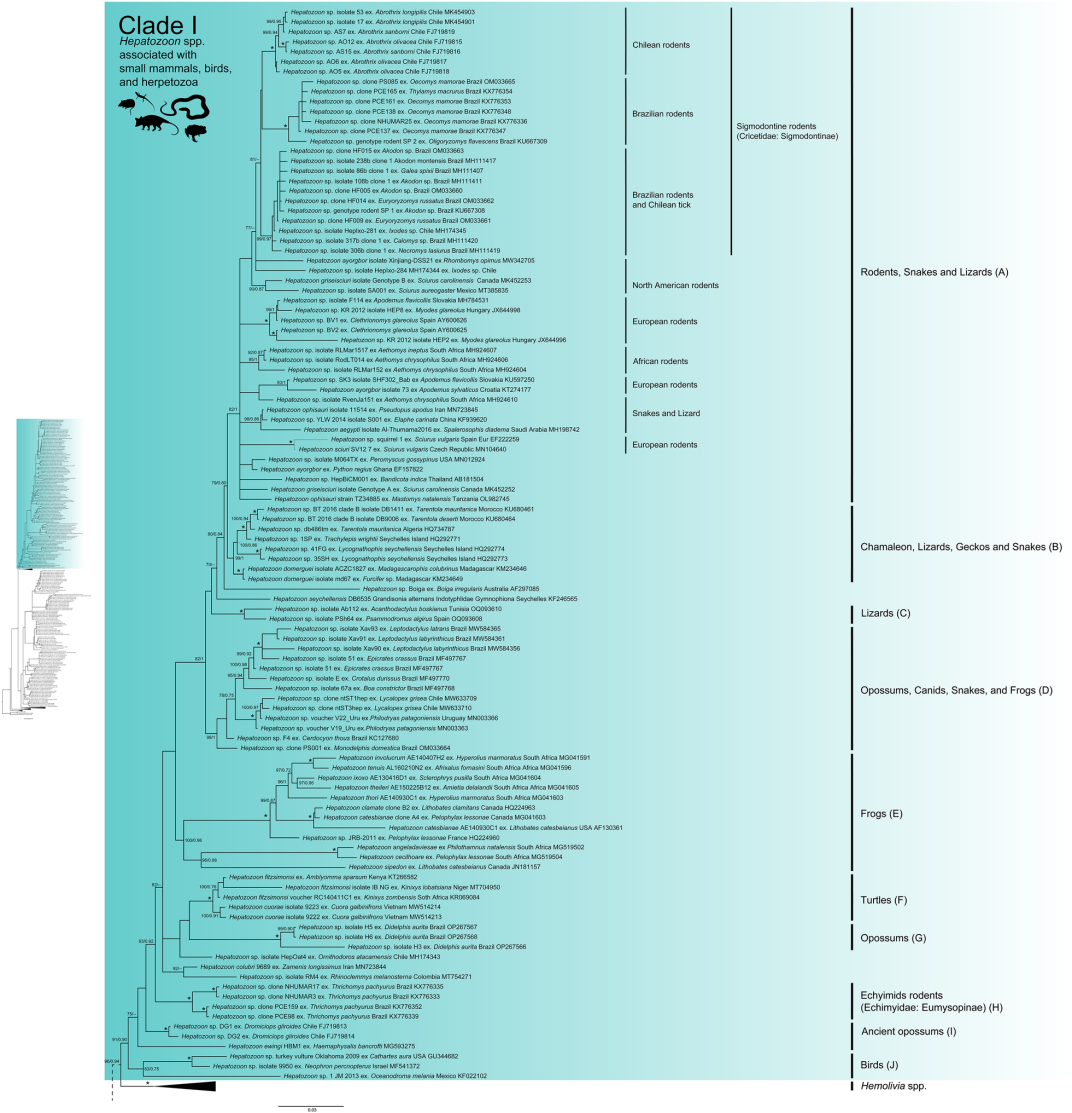
Phylogeny of *Hepatozoon* spp. associated with small mammals, birds, and herpetozoa (Clade I). Maximum likelihood (ML) and Bayesian inference (BI) 18S rRNA gene consensus tree constructed for a subset of Hepatozoon spp. using 241 sequences and an alignment of 2,001 bp. Best-fit evolutionary models calculated for ML and BI methods were TVM+F+G4 and M134, M85, M15, respectively. Ultrafast-bootstrap values and Bayesian posterior probabilities are indicated above or below each branch. Asterisks (*) indicate node support of 100/1 for ML and BI, respectively. GenBank accession numbers are located at the end of tip labels. The scale bar indicates the number of nucleotide substitutions per site. The phylogeny on the left represents a section of the complete *Hepatozoon* phylogeny. A dashed branch symbolizes the connection between this section and the remaining of the phylogeny

**Fig. 6:**
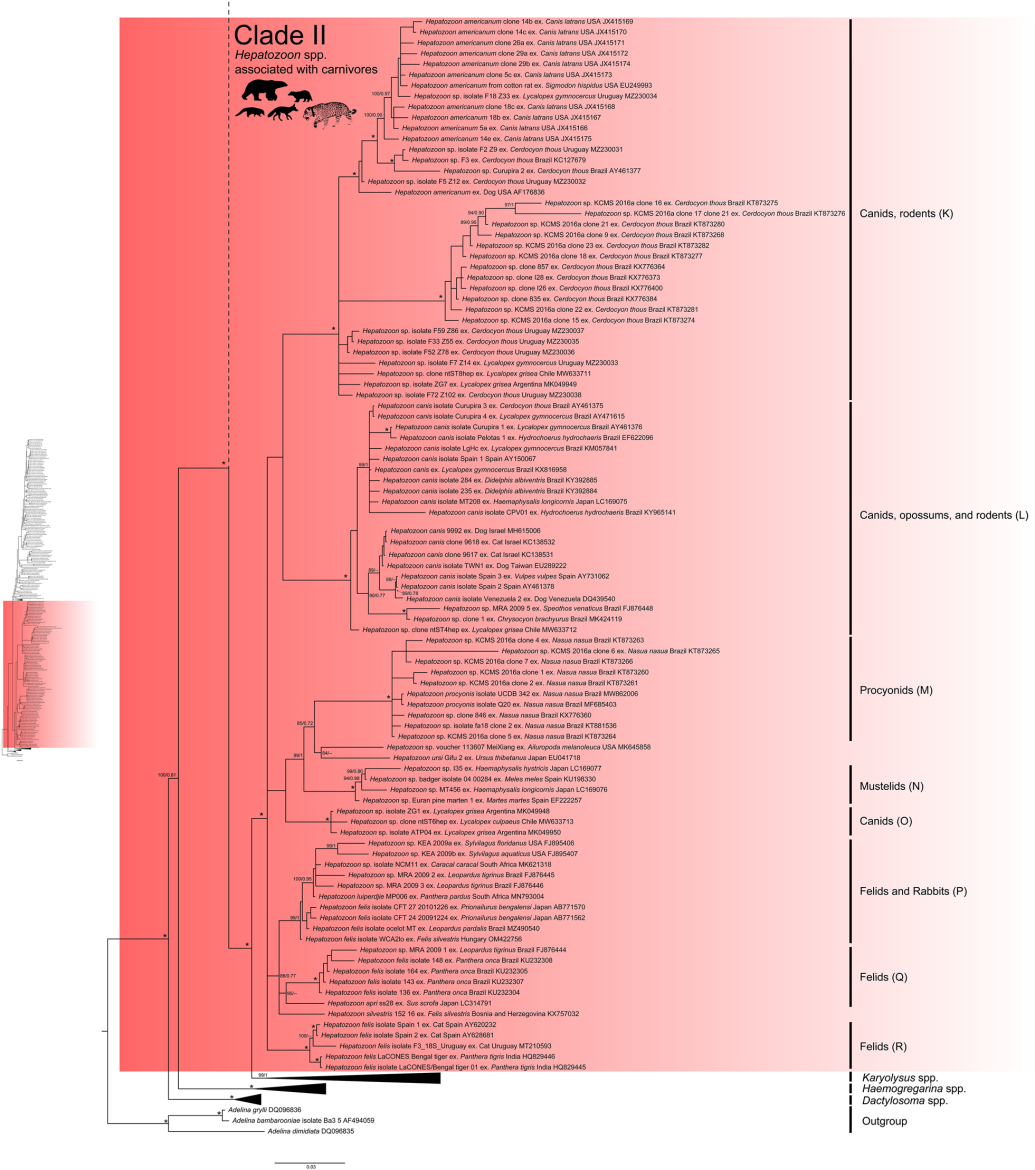
Phylogeny of *Hepatozoon* spp. associated with carnivores (Clade II). Maximum likelihood (ML) and Bayesian inference (BI) 18S rRNA gene consensus tree constructed for a subset of Hepatozoon spp. using 241 sequences and an alignment of 2,001 bp. Best-fit evolutionary models calculated for ML and BI methods were TVM+F+G4 and M134, M85, M15, respectively. Ultrafast-bootstrap values and Bayesian posterior probabilities are indicated above or below each branch. Asterisks (*) indicate node support of 100/1 for ML and BI, respectively. GenBank accession numbers are located at the end of tip labels. The scale bar indicates number of nucleotide substitutions per site. The phylogeny on the left represents a section of the complete *Hepatozoon* phylogeny. A dashed branch symbolizes the connection between this section and the remaining of the phylogeny

Both ML and BI trees revealed two main clades with strong node support: (i) *Hepatozoon* spp. associated with small mammals, birds, and herpetozoa (Fig. 5, Clade I), and (ii) *Hepatozoon* spp. related to carnivores (Fig. 6, Clade II). However, *Hemolivia* and *Karyolysus* render the genus *Hepatozoon* non-monophyletic (Figs. 5, 6). Previous studies also support these evolutionary relationships [120, 130, 134]. In the meantime, the paraphyly of the genus *Hepatozoon* remains unsolved, and a denser taxon sampling and additional molecular markers are required [124, 126, 134].

**Fig. 7:**
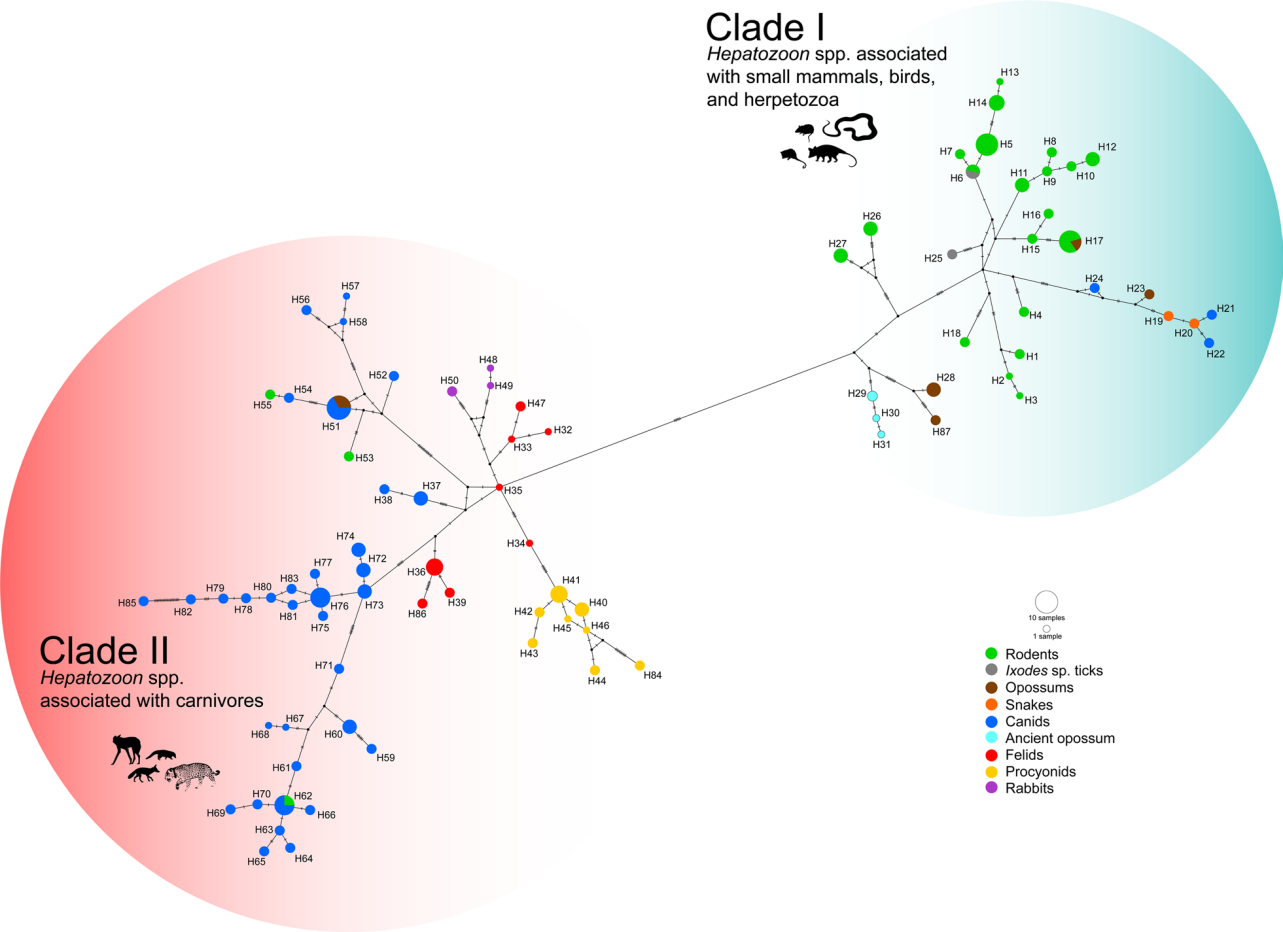
Haplotype network inferred for a subset of Hepatozoon 18S rDNA sequences obtained from wild mammals in the Americas using 115 sequences and an alignment of 621 bp. For details of haplotypes of *Hepatozoon* circulating among wild hosts refer to Table S4

The nucleotide alignment polymorphism analysis of *Hepatozoon* 18S rDNA sequences yielded 87 haplotypes (Fig. 7; Additional file 1: Table S4), Hd = 0.986 ± 0.002, with *π* = 0.04217 ± 0.00102, and *S* = 178. Moreover, the haplotype network showed a correspondence between haplogroups (Fig. 7) and the two major clades depicted in the phylogenies (Figs. 5, 6). Our haplotype network was similar to that reported by Perles et al. [109], which showed a bipartite split between haplotypes of (i) *Hepatozoon* spp. obtained from rodents and herpetozoa, and (ii) *Hepatozoon* spp. found in carnivores.

### *Hepatozoon* spp. Clade I

Clade I includes genotypes (Fig. 5) and haplotypes (Fig. 7) of *Hepatozoon* spp. recovered from mammals in the Americas belonging to the orders Carnivora (Canidae), Didelphimorphia (Didelphidae), Rodentia (Cricetidae, Echimyidae, and Sciuridae), and Microbiotheria (Microbiotheriidae). Although Clade I includes *Hepatozoon* spp. associated with small mammals, birds, and herpetozoa, two genotypes of *Hepatozoon* (MW633709, MW633710), found in South American gray foxes (*Lycalopex grisea*) in Chile [88], clustered with *Hepatozoon* genotypes associated with Patagonia green racer snakes (*Philodryas patagoniensis*) [135] (Fig. 5, subclade D). In addition, our BLASTn comparisons for these genotypes revealed a similarity of 99.84–99.68% with *Hepatozoon* genotypes recovered from Patagonia green racer snakes in Uruguay (99–100% query cover, 0 gaps, 0 E-value) [135]. Additionally, these associations were supported by the haplotype network (Fig. 7, Clade I, haplotypes H22 and H21).

Likewise, one *Hepatozoon* genotype (KC127680) characterized in a crab-eating fox (*Cerdocyon thous*) [97] and another genotype (OM033664) from a gray short-tailed opossum (*Monodelphis domestica*) [136] in Brazil, cluster with *Hepatozoon* genotypes related to snakes (Fig. 5, subclade D). Additionally, the haplotype network places H24 and H23 haplotypes on the same branch as H19 and H20 (Fig. 7, Clade I), which suggests a common parasitic pathway.

Our results collectively reveal that the *Hepatozoon* genotypes found in South American gray foxes in Chile, crab-eating fox, and gray short-tailed opossum in Brazil could have been acquired by preying on infected snakes [137–140]. However, rodents that frequently constitute the primary diet of both South American gray foxes [137, 141] and crab-eating foxes [140] may act as paratenic hosts for *Hepatozoon* species infectin snakes and lizards [142]. Moreover, crab-eating foxes also prey on frogs [143], which are also considered paratenic hosts for *Hepatozoon* spp. of reptiles [15]. However, infective cystic stages have been found in rodents from Brazil, suggesting that they are paratenic hosts in the transmission of *Hepatozoon* towards predators [144, 145].

A *Hepatozoon* genotype (KX776354) recovered from a Paraguayan fat-tailed mouse opossum (*Thylamys macrurus*) in Brazil [145] clustered into the clade of *Hepatozoon* spp. detected in rodents (Fig. 5, subclade of Brazilian rodents). This opossum species has been reported to be in syntopy with several rodent species, such as *Oecomys mamorae* [146], in which *Hepatozoon* has been also documented [136, 145], a fact that would explain a common haplotype (H17) among rodents and opossums (Fig. 7, Clade I). Therefore, it is likely that these small mammal species may share ectoparasites that could facilitate accidental cross-species infections [145].

*Hepatozoon* lineages found in South American cricetids (Rodentia: Cricetidae) and echimyids (Rodentia: Echimyidae) formed two clearly separated clades (Fig. 5, subclade sigmodontine rodents and subclade H). In this context, the *Hepatozoon* sp. infecting the Paraguayan punaré (*Thrichomys pachyurus*) appears to be a lineage specific to echimyids, as suggested for South American cricetid rodents [79, 102]. Likewise, the haplotype network supports the separation of *Hepatozoon* spp. from echimyids (Fig. 7, haplotypes H27 and H26). However, the inclusion of more *Hepatozoon* genotypes associated with South American echimyid rodents within this phylogenetic framework is needed to substantiate this phylogenetic hypothesis.

Regarding opossums, although the phylogeny does not relate *Hepatozoon* genotypes associated with the monito del monte (*Dromiciops gliroides*) and the big-eared opossum (*Didelphis aurita*) (Fig. 5, subclades G and I), the haplotype network places the haplotypes characterized from these opossum species at the same origin node (Fig. 7, haplotypes H29, H30, H31, H28, and H87). These findings suggest *Hepatozoon* lineages with a genetic structure associated with South American opossums. However, to further support this hypothesis, additional data are needed.

### *Hepatozoon* spp. Clade II

Clade II of *Hepatozoon* is composed of genotypes and haplotypes related to mammals of the orders Carnivora (Canidae and Felidae), Didelphimorphia (Didelphidae), Lagomorpha (Leporidae), and Rodentia (Cricetidae and Caviidae) (Fig. 6 and Fig. 7). Although this clade primarily comprises *Hepatozoon* spp. that infect carnivores, the inclusion of *Hepatozoon* genotypes related to Didelphimorphia, Lagomorpha, and Rodentia suggest that they correspond to *Hepatozoon* spp. that infected carnivores via predation [44, 45, 58, 59, 147]. This hypothesis would explain the shared haplotypes among canids and opossums (H51), and the clustering of rodent haplotypes with canids (Fig. 7, Clade II, haplotypes H55, H53, and H62).

On the other hand, experimental studies have shown that rabbits (Lagomorpha) serve as paratenic hosts for *Hepatozoon* spp. that infect carnivores [44]. But Allen et al. [59] proposed that some undescribed species of *Hepatozoon* may cycle in lagomorphs. However, the initial claim is supported by the close evolutionary relationships observed in *Hepatozoon* genotypes found in rabbits and felids (Fig. 6, subclade P), as well as the shared node of origin among rabbit and felid haplotypes (Fig. 7, Clade II, haplotypes H50, H48, H49, H33, H32, and H47). These associations could suggest that rabbits might serve as paratenic hosts for *Hepatozoon* spp. related to felids.

Regarding *Hepatozoon* genotypes and haplotypes of Didelphimorphia and Rodentia, their clustering into Clade II suggests that the sampled mammals came from regions endemic for canine hepatozoonosis; moreover, it demonstrates the potential of these mammalian groups to act as paratenic hosts for *Hepatozoon* species that infect canids [148–150]. Particularly, rodents have been implicated as vertebrate reservoirs of *Hepatozoon* in wildlife [15, 147]. Indeed, infective cystic stages in rodents facilitate the persistence of *Hepatozoon* spp. in the ecosystem [144, 147], including *H. americanum* [45]. This fact might account for the shared genotypes or haplotypes among canid and rodents, for both *H*. *americanum* (Fig. 6, subclade K; and Fig. 7, haplotype H62) and *H*. *canis* (Fig. 6, subclade L; and Fig. 7, haplotypes H53 and H55). Furthermore, it could suggest that rodents might also act as paratenic hosts for *H*. *canis* in Brazil [148]. However, this hypothesis remains unclear [109, 144].

Overall, our results reveal previously unreported associations between *Hepatozoon* genotypes in both distantly related taxa (Fig. 5, subclade D; and Fig. 6, subclades K, L, and P) and closely related mammalian groups (Fig. 5, subclades Sigmodontine and North American rodents), as well as in predator–prey relationships (Fig. 5, subclade D; and Fig. 6, subclades K, L, and P). This wide host spectrum suggests that the diversity and biogeographical patterns of *Hepatozoon* spp. in wild mammals across the Americas are more complex than currently understood. In this sense, the studies of Di Cataldo et al. [88] and Weck et al. [136] may not accurately reflect the relationships among *Hepatozoon* genotypes found in foxes in Chile and fat-tailed mouse opossum in Brazil due to lack of data in their phylogenetic framework. Therefore, incorporating more *Hepatozoon* sequences into an alignment could support with more confidence the monophyly of new genotypes and clarify their evolutionary relationships and ecological associations, as our analyses demonstrate.

### Factors driving the diversity and transmission of *Hepatozoon* spp. in mammals of the Americas

The genetic diversity of a given species is modulated by multiple processes that include mutation, recombination, and biodemography [151, 152]. For *Hepatozoon* spp., the life cycle, transmission dynamics, and dispersion capacity are factors that shape their diversity as well [38]. Moreover, the transmission of *Hepatozoon* species may be facilitated by syntopy of hosts sharing ectoparasites [145], the distribution of suitable vectors [52, 62, 63, 153], and through ingestion of infective cystozoites by carnivorism [44, 45, 142] or ectoparasites attached to prey [18, 45]. However, it seems that host specificity and food webs play a crucial role in the transmission of *Hepatozoon* species, as corroborated by previous studies [147, 154].

Small mammals and herpetozoa are typical prey of carnivores, and those harboring cysts are likely to transmit *Hepatozoon* spp. to their predators. This ecological pattern is shown in Fig. 5, where occasional infections of canids with snake-related genotypes of *Hepatozoon* can be observed (subclade D), suggesting a low host specificity in certain *Hepatozoon* spp. However, given that vector capacity—defined as the daily rate of effective infections spread by a specific arthropod population and influenced by vector density, behavior, longevity, vector-host encounter rates, and vector competence—varies among invertebrate hosts [155–158], predators can be considered dead-end hosts for these *Hepatozoon* spp., thus affecting the transmission dynamics. In this context, predators may contribute to reducing the transmission of the parasite (dilution effect) [41, 147]. Nevertheless, these infections may affect the immune response of predators, potentially increasing their susceptibility to other infectious agents [93, 159, 160].

In addition, *Hepatozoon* spp. exhibit greater host specificity in invertebrates (definitive host) than in vertebrates (intermediate host) [15, 17, 123]. Therefore, the degree of specificity that an invertebrate parasite shows to its vertebrate host will define the chance for a given *Hepatozoon* sp. to find and colonize a suitable vertebrate host [157, 161]. Colonization may be affected by the immune response of the vertebrate hosts [8]. Thus, considering that the composition of parasite communities is primarily structured by host species (both intermediated and definitive), phylogenetically related hosts are more likely to share parasite species since they exhibit similar immunological pathways and ecological and evolutionary processes [8, 162]. These interactions with invertebrate and vertebrate hosts play a significant role in the diversification and dissemination of *Hepatozoon* spp.

Some studies have proposed that *Hepatozoon* genotypes exhibit close phylogenetic relationships and a genetic structure according to the vertebrate groups that they parasitize [109, 144, 163]. Notably, rodent-associated *Hepatozoon* spp. seem to be specific, in contrast to those species that infect reptiles [164]. Likewise, studies have confirmed a degree of genetic diversity in *Hepatozoon* spp. infecting rodents [144, 145]. Although some genotypes of *Hepatozoon* found in rodents are shared with reptiles, they are considered to be *Hepatozoon* spp. of reptiles using rodents as paratenic hosts within their life cycle [142].

In particular, the findings in Chile suggest discrete lineages of *Hepatozoon* spp. associated with the native rodent genera *Abrothrix, Oligoryzomys*, and *Phyllotis* [75, 79, 102, 132], and with the ancient marsupial monito del monte (*D. gliroides*) [75], suggesting that *Hepatozoon* co-evolved with these mammals [102, 165, 166]. Thus, the evolutionary history and diversification dynamics of these hosts could be shaping the phylogenetic relationships and genetic structure of the *Hepatozoon* lineages characterized in Chile [166]. However, a denser sampling across hosts and the inclusion of both 18Sr DNA and *cox1* sequences in the genetic analyses are needed to support this hypothesis.

The knowledge of the *Hepatozoon* genotypes and haplotypes circulating among wildlife mammals in the Americas provides valuable insight into the epidemiology of this hemoparasite, shedding light on exposed or susceptible hosts. Also, it facilitates tracking the spread and occurrence of unique haplotypes of *Hepatozoon* among mammalian groups (Fig. 7), especially in threatened host species. Although a greater diversity of *Hepatozoon* spp. is found in canids and rodents, this may reflect a sampling bias. Overall, these results reveal a significant lack of data and highlight the need for a comprehensive sampling of less prospected mammals in the Americas.

### Would *Hepatozoon* spp. pose a risk to mammals of the Americas?

Infectious diseases pose a significant threat to wildlife, leading to population decline, biodiversity loss, ecological disruptions, and an increased risk of disease transmission [11, 12, 167]. Therefore, studying infectious diseases in wildlife is crucial for the conservation of animal biodiversity and human health, particularly in the context of global climate change [3, 168]. In the Americas between 1916 and 2022, *Hepatozoon* spp. have been reported in 107 species of mammals, belonging to 62 genera, 18 families, and nine orders (Table S1). This fact highlights the broad range of susceptible mammals and the potential impact on health that *Hepatozoon* spp. in the wildlife of the Americas could pose.

Based on our findings, *H. canis* and *H. felis* emerge as the most widespread species among wild mammals in the Americas. *Hepatozoon canis* was identified in 13 wild mammal species, infecting 133/657 individuals (20.24%), while *H*. *felis* was found in 12 species, infecting 132/475 individuals (27.97%) (Additional file 1: Table S1). It is recognized that *H. canis* infection in canids might persist sub-clinically, and its severity can range from mild to life-threatening [16, 53, 66, 83], while *H. felis* seems well suited to felid hosts [68]. However, IFs of *H. canis* and *H. felis* among non-canid and non-felid hosts (Additional file 1: Table S1) raise concerns and warrant further investigation because of the potential risks they might pose to other mammalian groups, particularly those with conservation threats [169].

Remarkably, *H. americanum* exhibited IFs of 44.09% (41/93 individuals) across three wild mammal species, while *H. americanum*-like showed IFs of 11.43% (44/385 individuals) in five canid species (Additional file 1: Table S1). The first species is considered the primary cause of American canine hepatozoonosis (ACH) in North America [16, 38, 66, 170]. Meanwhile, the latter is a species closely related to *H*. *americanum*, and is emerging in South American canids [13, 67, 171]. Although *H*. *americanum* was found infecting only two mammal species in North America, it showed higher IFs.

While *H. americanum* and *H. americanum*-like were found in one North American and four South American canid species, respectively, *H. canis* was found in nine species of canids, including three in North America and six in South America (Additional file 1: Table S1). Additionally, *H*. *felis* was found in three canid species and six felid species across the Americas (Additional file 1: Table S1). Further assessment is necessary to understand the spread and potential impact of these infections among canid and felid populations, given that carnivores play an essential role in maintaining balance of the ecosystems as predators within the food webs, and are threatened species, [172–177].

The high occurrence of *Hepatozoon* infection in both North American and South American canids can be linked to their presence in endemic areas for canine hepatozoonosis [18, 58, 59, 144, 153]. It is worth mentioning that some canids, such as the coyote (*Canis latrans*) and crab-eating fox, are expanding beyond their natural ranges [178]. This fact, coupled with cross-breeding events with domestic dogs [178, 179], poses a significant risk for spillover of parasites between domestic animals and wildlife, as well as the emergence of new endemic canine hepatozoonosis foci [180].

Indeed, coyotes are commonly reported to be infected with *H. americanum* in North America [87, 153, 181–184], while crab-eating foxes are associated with *H*. *canis* and *H. americanum*-like infections in South American ecosystems [67, 171, 185, 186]. Both *H*. *americanum* and *H. canis* are recognized for their virulence in canids [16, 66, 84], as well as for their diverse modes of transmission between hosts and ticks, that are common ectoparasites of canids in the Americas [52, 63, 187].

Notably, *Hepatozoon* was primarily observed in Carnivora (42.12%; 730/1,733); however, Rodentia exhibited the highest number of species (62.26%; 66/106) infected with *Hepatozoon* (Additional file 1: Table S1). This can be attributed to the diverse ecological traits of rodents, such as their fast life pace, terrestriality, high population densities, various activity cycles, and diet breadth [188]. Likewise, being one of the most geographically widespread, diverse, and abundant mammalian orders, Rodentia influences parasite richness and transmission [3, 21, 189]. Our findings highlight the importance of both carnivores and rodents to understanding the epizootiology and transmission of *Hepatozoon* spp. Moreover, they suggest that rodents are key in maintaining *Hepatozoon* spp. in the ecosystems of the American continent.

Despite the fact that certain etiological agents in wildlife may not cause diseases in their hosts due to a harmonious parasite–host relationship (co-evolution) [190, 191], it is worth mentioning that while *Hepatozoon* infections in wildlife are often subclinical, they may vary from mild to severe [67, 182, 183, 192]. Indeed, these hemoparasites become pathogenic and opportunistic in immunocompromised individuals, exhibiting high virulence in concurrent infections, thereby increasing susceptibility to other vector-borne agents [66, 181, 193–195]. Furthermore, spillover of *Hepatozoon* spp. in atypical hosts may lead to infections more virulent than those observed in natural hosts [196–198].

*Hepatozoon* infections have been associated with mortality and clinical diseases in hyenas (*Crocuta crocuta*) and coyotes [183, 199, 200], with recent evidence linking the infection to myocarditis and myositis in coyotes [153]. In crab-eating foxes, *Hepatozoon* infections result in mild anemia, abnormal blood values, liver degeneration, and splenic growth [185]. For instance, in impalas (*Aepyceros melampus*), symptoms of mild hepatitis and lymphadenitis have been associated to *Hepatozoon*-like infection [201, 202]. Moreover, in rodents, high parasitemia in specific tissues (bone marrow, lung, and liver) post-second merogony [42] leads to anemia, fatigue, inflammation, and sometimes death [159, 203–206].

*Hepatozoon* transmission in vertebrates primarily occurs through the ingestion of an infected blood-sucking invertebrate. However, the second merogony—a phase of asexual reproduction producing cystozoites—seems pivotal for some *Hepatozoon* spp., as cystozoites are the infectious forms leveraging transmission through predation, thereby facilitating its spread across food webs [15, 42]. Rodents often act as paratenic hosts in this mode of infection [15, 147, 207, 208]. In fact, studies indicate that carnivores may acquire *Hepatozoon* infection by preying on rodents [147, 207]. Nevertheless, the role of rodents in the epidemiology of *Hepatozoon* species detected in carnivores is still unclear [145, 209].

Over 42,100 species are at risk of extinction, of which 11,367 (27%) are mammals [210]. Among 107 mammal species documented with *Hepatozoon* infections, 10 (9.35%) face potential extinction, two (20%) are vulnerable (VU), and eight (80%) are near threatened (NT); 86 (80.37%) are categorized as least concern (LC); three (2.80%) have data-deficient status (DD), and nine (8.41%) lack available data. Of the species positive for *Hepatozoon*, 49 (45.79%) have stable populations, 22 (20.56%) are decreasing, seven (6.54%) are increasing, 22 (20.56%) have unknown population status, and nine (8.41%) lack accessible data (see Additional file 1: Table S1).

A pre-existing infection can aid the establishment of a new infectious agent which otherwise might have been cleared by the host’s immune system; consequently, the acquisition of a novel infectious agent may promote the dissemination of an existing latent or dormant infection in the host, acting synergistically to enhance pathogenic processes, parasite transmission, and disease severity [93, 159, 160].

*Hepatozoon* infection in mammals of the Americas requires comprehensive attention, considering factors such as the availability of suitable arthropod vectors, the prevalence of infection in intermediate hosts, and the susceptibility of different mammal species. In addition, it is important to recognize other threats to wildlife in the Americas, including habitat loss (by urbanization, livestock, and farming), pollution, climate change, and tick-borne diseases [156, 211]. The conjunction of these factors may influence the spillover of *Hepatozoon* infections among wild and domestic animals. To date, only one study has monitored the fluctuation and assessed the impact of *Hepatozoon* infection on health in wildlife of the Americas [89], for which additional studies of this kind are necessary.

Given that parasites pose a risk to species susceptible to extinction [212], detecting and monitoring *Hepatozoon* spp. in wildlife becomes necessary. Genetic screening can reveal the patterns of dispersion of *Hepatozoon* lineages among hosts, enriching our knowledge of their roles in the *Hepatozoon* epizootiology [169, 212, 213]. It is crucial for further research and surveillance to comprehensively understand the dynamics of *Hepatozoon* infections in mammal populations of the Americas and assess their potential as a significant threat [88].

## Conclusions and future perspectives

This review provides valuable insight into the distribution of *Hepatozoon* among mammalian hosts and potential vectors in the Americas, establishing a foundation for subsequent research. Notably, this review represents the first comprehensive summary of *Hepatozoon* infection in wild mammals in the region. However, numerous questions remain unanswered, particularly regarding the impact of hemoparasites on the health of wild mammals in the Americas.

Given the expanding distribution of certain canids, combined with hybridization events between some species and the diverse transmission modes of *Hepatozoon*, there is a risk for spillover between wildlife and domestic animals. This could also lead to the emergence of new areas with endemic foci for canine and felid hepatozoonosis. Canid species such as coyotes, crab-eating foxes, South American gray foxes, and Pampas foxes may serve as effective sentinels to track the expansion of *H. americanum*, *H. canis*, *H. americanum*-like, and *H. felis* in ecosystems across the American continent. Finally, genomic data and new molecular markers are urgently needed for the implementation of effective strategies to detect, control, and manage *Hepatozoon* infections.

### Supplementary Information

**Additional file 1:** **Table S1.** Wild mammals with reported *Hepatozoon* spp. in the Americas.**Table S2.** Primer details and thermal conditions used for the molecular diagnosis and genotyping of *Hepatozoon* in wild mammals of the Americas.**Table S3.** 18S rDNA *Hepatozoon* sequences (≥387 bp) listed in GenBank database until December 2022, flanking one or more of the nine SSU rRNA regions.**Table S4.** Genetic variants of *Hepatozoon* spp. circulating among wild mammals in the Americas.

## Data Availability

The datasets generated and analyzed in this review are available in the Additional file 1 (Tables S1, S2, S3, and S4).
