# Correction: *Hepatozoon* (Eucoccidiorida: Hepatozoidae) in wild mammals of the Americas: a systematic review

**DOI:** 10.1186/s13071-024-06309-2

**Published:** 2024-05-16

**Authors:** Richard Thomas, Adriana Santodomingo, Liliana Saboya-Acosta, Julian F. Quintero-Galvis, Lucila Moreno, Juan E. Uribe, Sebastián Muñoz-Leal

**Affiliations:** 1 Departamento de Ciencia Animal, Facultad de Ciencias Veterinarias, Universidad de Concepción, Chillán, Chile; 2 Pontificia Universidad Javeriana, Facultad de Estudios Ambientales y Rurales, Doctorado en Estudios Ambientales y Rurales, Carrera 7 N 40-62, Bogotá, Colombia; 3 Instituto de Ciencias Ambientales y Evolutivas, Universidad Austral de Chile, Valdivia, Chile; 4 Millenium Nucleus of Patagonian Limit of Life (LiLi), Valdivia, Chile; 5 Departamento de Zoología, Facultad de Ciencias Naturales y Oceanográficas, Universidad de Concepción, Concepción, Chile; 6 Departamento de Biodiversidad y Biología Evolutiva, Museo Nacional de Ciencias Naturales (MNCN-CSIC), Madrid, Spain

**Correction: Parasites & Vectors (2024) 17:108** 10.1186/s13071-024-06154-3

Following publication of the original article [1], a number of inaccuracies came to the attention of the authors. The original description of the *Hepatozoon* genus had been attributed to Balfour, 1905, whereas the correct ascription is to Miller, 1908, who established the *Hepatozoon* genus [2]. Balfour described the type species, *Hepatozoon muris* (syn. *Leucocytozoon muris* Balfour, 1905; *Hepatozoon perniciosum* Miller, 1908), which latter passed to *Hepatozoon* genus after the reorganization of hemogregarines by Wenyon in 1926 [3].

In addition, throughout the text as well as in Fig. 5, Fig. 6, and Supplementary Tables S1 and S4, the Pampa’s fox (*Lycalopex gymoncercus*) was mentioned to have distribution in Chile and southern Argentina, but this is not correct: the species corresponds to the South American grey fox (*Lycalopex grisea*) [4]. Moreover, the sequence MZ230033 in Fig. 5 should be associated with Uruguay, not Chile, as it previously was.

These amendments to the published article alter the values of *Hepatozoon* infection in American canids throughout the review and also imply that preys such as snakes can vehicle *Hepatozoon* spp. to infect South American grey foxes [5–7]. Following these amendments, the total number of species of mammals that have tested positive for *Hepatozoon* infection has been updated to 107, and the infection statistics among these species, including those at risk of extinction and their conservation statuses, has been updated. The original article has been updated to amend these errors.
